# Multiple remote C(sp^3^)–H functionalizations of aliphatic ketones *via* bimetallic Cu–Pd catalyzed successive dehydrogenation†

**DOI:** 10.1039/d2sc05370e

**Published:** 2022-11-14

**Authors:** Hongyi Li, Chang Yin, Sien Liu, Hua Tu, Ping Lin, Jing Chen, Weiping Su

**Affiliations:** State Key Laboratory of Structural Chemistry, Center for Excellence in Molecular Synthesis, Fujian Science & Technology Innovation Laboratory for Optoelectronic Information of China, Fujian Institute of Research on the Structure of Matter, Chinese Academy of Sciences Fuzhou 350002 China chenjing@fjirsm.ac.cn wpsu@fjirsm.ac.cn; College of Chemistry and Materials Science, Fujian Normal University Fuzhou 350002 China

## Abstract

The dehydrogenation-triggered multiple C(sp^3^)–H functionalizations at remote positions γ, δ or ε, ζ to carbonyl groups of aliphatic ketones with aryl/alkenyl carboxylic acids as coupling partners have been achieved using a bimetallic Cu–Pd catalyst system. This reaction allows access to alkenylated isocoumarins and their derivatives in generally good yields with high functional group tolerance. The identification of bimetallic Cu–Pd synergistic catalysis for efficient successive dehydrogenation of aliphatic ketones, which overcomes the long-standing challenge posed by the successive dehydrogenation desaturation of terminally unsubstituted alkyl chains in aliphatic ketones, is essential to achieving this bimetallic Cu–Pd catalyzed dehydrogenation coupling reaction.

## Abstract 2

The dehydrogenation-triggered multiple C(sp^3^)–H functionalizations at remote positions γ, δ or ε, ζ to carbonyl groups of aliphatic ketones with carboxylic acids as coupling partners have been achieved using a bimetallic Cu–Pd catalyst system.

## Graphical abstract

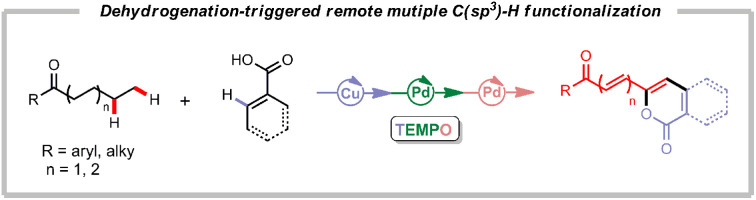

## Introduction

**Scheme 1:**
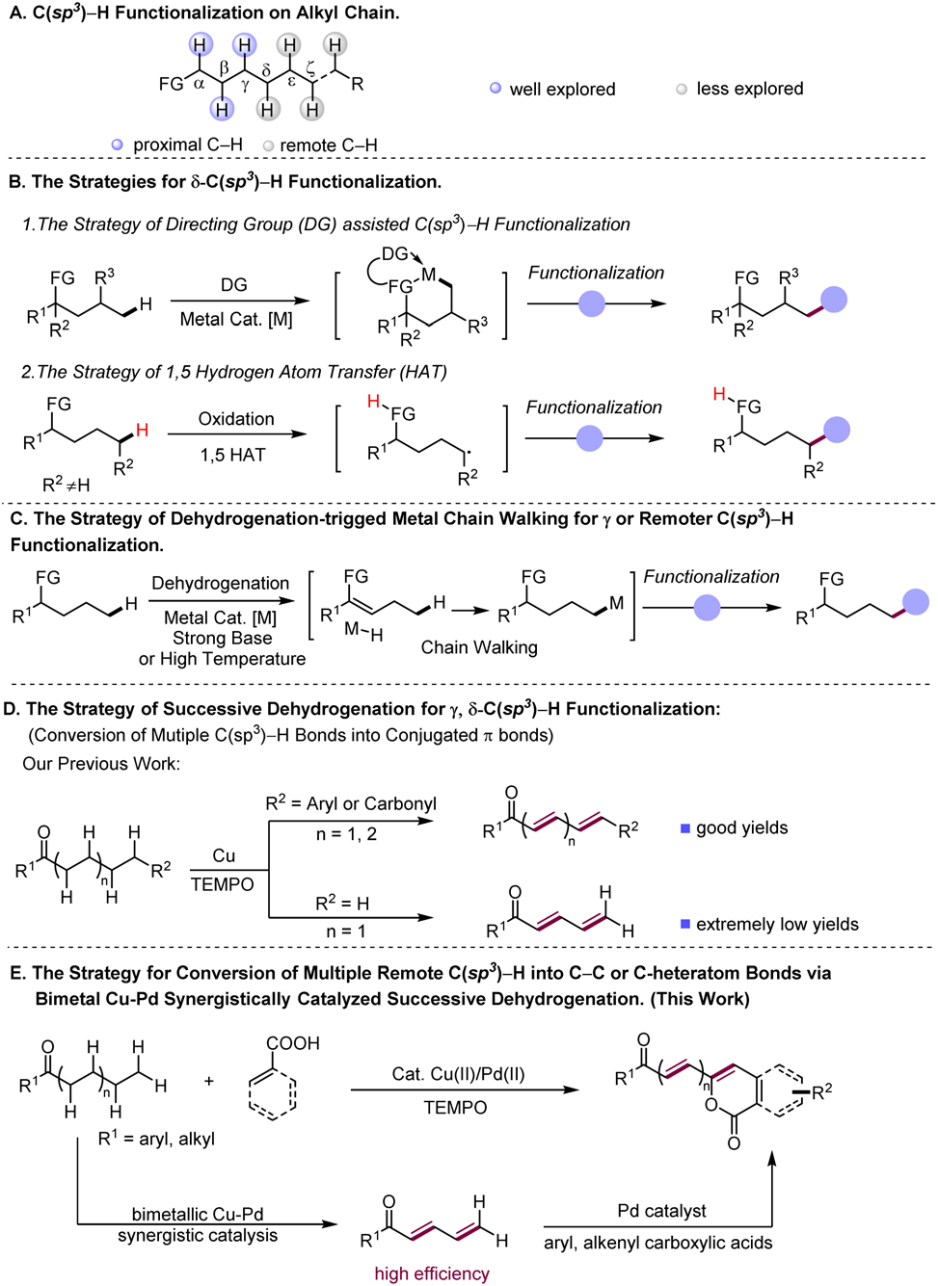
Development of the strategies for functionalization of remote C(sp^3^)–H bonds.

Transformations of simple chemicals into value-added organic compounds through selective functionalization of ubiquitous but inert C(sp^3^)–H bonds are of intense current interest.^1^ In general, the reactions at such C–H bonds need either the assistance of directing groups or the suitable electronic properties of reactants.^2^ In this regard, transformations based on activation of C(sp^3^)–H bonds proximal to functional groups, facilitated by either organic or transition metal catalysis, have been well explored in recent decades^3^ (Scheme 1A). In contrast, the examples of C(sp^3^)–H functionalization at the positions that are less reactive and distant from functional groups (FG) are scarce (Scheme 1A), indicative of the great challenge faced by chemists in this field. Taking advantage of appropriate transient directing groups, a handful of elegant methods for obtaining δ-C(sp^3^)–H functionalization of amines^4^ and γ-C(sp^3^)–H functionalization of carbonyl compounds^5,6^*via* six-membered metallacycles have been recently disclosed (Scheme1B(1)). However, linear aliphatic feedstocks are not amenable to these reactions. On the other hand, to aim at remote non-directed C(sp^3^)–H functionalization, the strategy of radical-induced hydrogen atom transfer (HAT) has been successfully developed, which enables remote C(sp^3^)–H halogenation^7*a*,*b*^ and intramolecular cyclization reactions^7^ (Scheme 1B(2)). By merging this HAT strategy with photocatalysis, selective γ or δ C(sp^3^)–H alkylation and arylation reactions have been achieved.^8^ As demonstrated in the reported reactions, such a HAT strategy works well for both secondary and tertiary C(sp^3^)–H bonds, but is difficult to realize functionalization of less active primary C(sp^3^)–H bonds.

Complementary to the prior arts in selective remote C–H functionalization,^9^ the development of new synthesis protocols that are able to override the space limit encountered by the existing methods, especially ones that operate for readily available substrates, would be highly desirable. The C–H functionalization reactions initiated by dehydrogenative desaturation have appeared as a promising tool for syntheses of complex molecular architectures.^10^ These reported strategies merge dehydrogenation of aliphatic compounds to generate reactive unsaturated intermediates with various alkene coupling approaches, such as conjugate addition,^11^ redox cascade^12^ and Diels–Alder reactions,^13^ and therefore give rise to diverse interesting chemical transformations, demonstrating the ability of this type of reaction to rapidly construct complex molecules from simple substrates *via* formal C(sp^3^)–H functionalization.^10*a*^ Interestingly, a few elegant studies have demonstrated that some of the dehydrogenation-induced C(sp^3^)–H functionalization reactions go through a so-called metal chain walk process which involves multiple metal-enabled β-H elimination to M-H and re-insertion of M-H into C–C double bonds towards formation of remote C(sp^3^)–H metalation intermediates, to achieve functionalization of remote C(sp^3^)–H bonds^14^ (Scheme 1C). Very recently, diverse types of metal-catalyzed methods have been developed for efficient dehydrogenative desaturation of saturated substrates to construct alkenes,^15,16^ which would further spur the growth of the tactics for dehydrogenation-triggered C(sp^3^)–H functionalization.

Recently, our lab disclosed a Cu-catalyzed successive dehydrogenation of terminal substituted aliphatic ketones to construct conjugated dienones or polyenones^17^ (Scheme 1D). This successive dehydrogenation sequence mechanistically involves the initial α, β-desaturation followed by further dehydrogenation desaturation of the resultant enone intermediate to conjugated dienone, implicating that the reactivity at α-carbon was transmitted to a distal position through multiple conjugated bonds. Inspired by our previous finding, we envisioned the feasibility of the remote C(sp^3^)–H functionalization of aliphatic ketones triggered by successive dehydrogenation desaturation, given that successive desaturation along the carbon chain could convert distal inert C(sp^3^)–H bonds into more reactive double bonds. However, three concerns remain to be addressed for the successful realization of remote functionalization protocols based on successive desaturation. Although the successive dehydrogenation of terminal substituted ketones indeed occurs, our previous method affords extremely low yield in successive desaturation of the aliphatic acyclic ketones lacking terminal substituents (Scheme 1D). How to increase the efficiency of successive dehydrogenation for terminally unsubstituted ketones is the first concern. Secondly, conjugated dienones and polyenones, which are the proposed intermediates in the designed reaction, are the reactive intermediates prone to polymerization and olefin oxidation.^18^ Thirdly, the compatibility between dehydrogenative desaturation and the designed alkenyl coupling process is required.

In nature, natural product formation occurs through a metabolic network that operates by integrating multi-enzyme-catalyst systems.^19^ In the case of enzyme-catalyzed alcohol oxidation,^20^ the dehydrogenation sequence of alcohol relies on synergic action of two individual enzymes, namely alcohol desaturase and aldehyde desaturase enzymes, to transform alcohol into acetic acid in tandem. Inspired by nature, we hypothesized that a suitable bimetallic catalyst system may efficiently catalyze successive dehydrogenation of long alkyl chains in aliphatic ketones to polyenones through the synergistic catalysis of two metallic catalysts to overcome the low-efficiency issue in previously reported successive dehydrogenation reactions, and meanwhile promote rapid coupling of polyenone intermediates with the other coupling partner to avoid the detrimental side-reaction of reactive polyenone intermediates. Herein, we validate the feasibility of this hypothesized reaction and report a bimetallic Cu–Pd synergistically catalysed^21^ remote arylation or alkenylation of aliphatic acyclic ketones with aryl/alkenyl carboxylic acids as coupling partners (Scheme 1E), which proceeds *via* the bimetallic Cu–Pd synergistically catalyzed successive dehydrogenation of aliphatic ketones to generate conjugated dienone or polyenone intermediates, and subsequent Pd-catalyzed carboxyl directed *ortho*-C–H olefination of aryl/alkenyl carboxylic acids with ployenone intermediates.

## Results and discussion

With the considerations regarding synergistic bimetallic catalysis in mind, we investigated the reaction of valerophenone (1a) with *ortho*-methoxy benzoic acid (2a) in the presence of various metal catalysts (Table 1). Benzoic acids were used as arylating reagents because benzoic acids are widely available and versatile arylating reagents through carboxyl-directed *ortho*-functionalization reactions or decarboxylative cross-coupling reactions.^22^ A variety of metal catalysts regularly used for promoting C(sp^2^)–H functionalizations were investigated in combination with a catalytic amount of Cu(OAc)_2_ and a stoichiometric amount of TEMPO, which showed that most metal catalysts did not give the targeted arylation product. To our delight, Pd(OAc)_2_, a catalyst that was previously used for dehydrogenation reactions of saturated carbonyl compounds in the presence of a strong base,^10*b*^ was found to enable the expected ketone δ-arylation reaction to occur and furnish alkenylated-isocoumarin (3a) as a final product (see the ESI† for details). Isocoumarins are an important class of bioactive compounds^23^ and also versatile starting materials for organic synthesis, and alkenylated-isocoumarin (3a) possesses multi-function handles for further downstream diversification,^18^ which implicates that this method for synthesis of alkenylated-isocoumarin has the significant potential for synthetic applications. Encouraged by these initial results, we performed optimization studies on this dehydrogenation-triggered coupling of 1a with 2a in detail. After extensive screening of reaction parameters, we discovered that the use of Pd(OAc)_2_(10 mol%)/SIPr (L1)(15 mol%)/Cu(OAc)_2_ (20 mol%) as a bimetallic catalyst system, 4-OMe-TEMPO (4 equiv.) as an oxidant, and Zn(TFA)_2_·H_2_O (10 mol%) as a promoter gave rise to efficient reaction of 1a (0.24 mmol, 1.2 equiv.) with 2a (0.2 mmol) at 130 °C in toluene (2.5 mL) with an 81% isolated yield of 3a (Table 1). The early trials and control experiments revealed that Pd(OAc)_2_ was indispensable to the reaction (entry 1). Other Pd salts were found to be inferior to Pd(OAc)_2_ (entries 2 and 3). Among the ligands examined, SIPr (L1) was optimal probably due to its strong σ donor and high bulkiness, which may stabilize Pd species and also promote reoxidation of Pd(0) intermediates in oxidative C–H activation reactions.^24^ Phosphine ligands had a beneficial effect on the reaction but were less efficient than L1 due to facile oxidation to phosphine oxides^25^ (entries 4 and 5). The target product cannot be detected in the absence of Cu(OAc)_2_, indicating that the copper salt played an indispensable role in the reaction (entry 6). A catalytic amount of Zn(TFA)_2_·H_2_O significantly enhanced the reaction efficiency (entry 7), which is likely due to promoting the enolization step *via* formation of a Lewis acid–base adduct.^26^ Apparently, 4-OMe-TEMPO is an optimal oxidant, since other TEMPO derivatives such as TEMPO and 4-NHAc-TEMPO were found capable of facilitating the desired cascade reaction, but relatively less effective (entries 8 and 9). It is worth mentioning that using other common oxidants instead of 4-OMe-TEMPO did not work in this reaction (see the ESI† for details). Screening different solvents demonstrated that aromatic solvents were optimal, though 1,4 dioxane also delivered the product in 40% yield (entry 11,12). Concentration was also an important factor influencing reaction outcomes; running the reaction at a higher concentration led to decomposition of active intermediates, while a lower concentration influenced the kinetics of enone formation^11*d*,16*f*^ (entries 13–15).

**Table 1:** Optimization studies for bimetallic Cu–Pd catalyzed remote C(sp^3^)–H arylation of alkyl aryl ketones with aryl carboxylic acids^a^

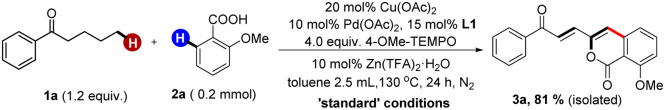		
Entry	Variation from the standard conditions	Yield (%) of 3a^b^
1	Without Pd(OAc)_2_	n.d.
2	Pd(TFA)_2_ instead of Pd(OAc)_2_	72%
3	Pd(PPh_3_)_4_ instead of Pd(OAc)_2_	58%
4	L2–L9 instead of L1	Listed below
5	Without L1	31%
6	Without Cu(OAc)_2_	n.d.
7	Without Zn(TFA)_2_·H_2_O	44%
8	Without TEMPO	n.d.
9	TEMPO instead of 4-OMe-TEMPO	70%
10	4-NHAc-TEMPO instead of 4-OMe-TEMPO	76%
11	Solvent = 1,4 diene	40%
12	Solvent = PhCF_3_	73%
13	2 mL toluene was used	75%
14	1 mL toluene was used	28%
15	3 mL toluene was used	77%
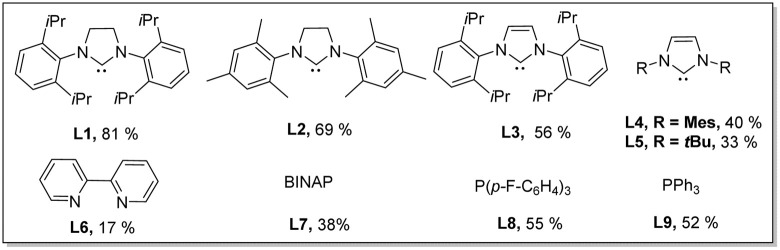		

a Reaction conditions: 1a (0.24 mmol, 1.2 equiv.), 2a (0.2 mmol).

b Yields are the isolated yields. n.d., not detected.

With the optimized catalytic system in hand, the scope of carboxylic acid was investigated for the dehydrogenation-triggered remote functionalization of aliphatic acyclic ketones. As show in Table 2, carboxylic acids bearing various functional groups regardless of electron-donating or -withdrawing ones, such as halides (3d, e), CF_3_ (3f), COPh (3j), NMe_2_ (3k), and CO_2_^*t*^Bu (3o), all gave δ-arylated ketones in generally good yields. Notably, the base sensitive groups OAc (3g) and NHCO^*t*^Bu (3h) were also compatible due to the acidic reaction conditions. Other aromatic carboxylic acids, such as 1-naphthoic acid (3p) and 1-benzothiophene-3-carboxylic acid (3q), were suitable substrates. Interestingly, this protocol allowed using alkenyl carboxylic acids (3r–3u) as coupling partners to furnish polyene structures which are frequently found in drugs and pharmaceutical intermediates,^27^ further highlighting the generality of this protocol.

**Table 2:** The scope of carboxylic acids for bimetallic Cu–Pd catalyzed remote C(sp^3^)–H functionalization of aliphatic acyclic ketones^a^

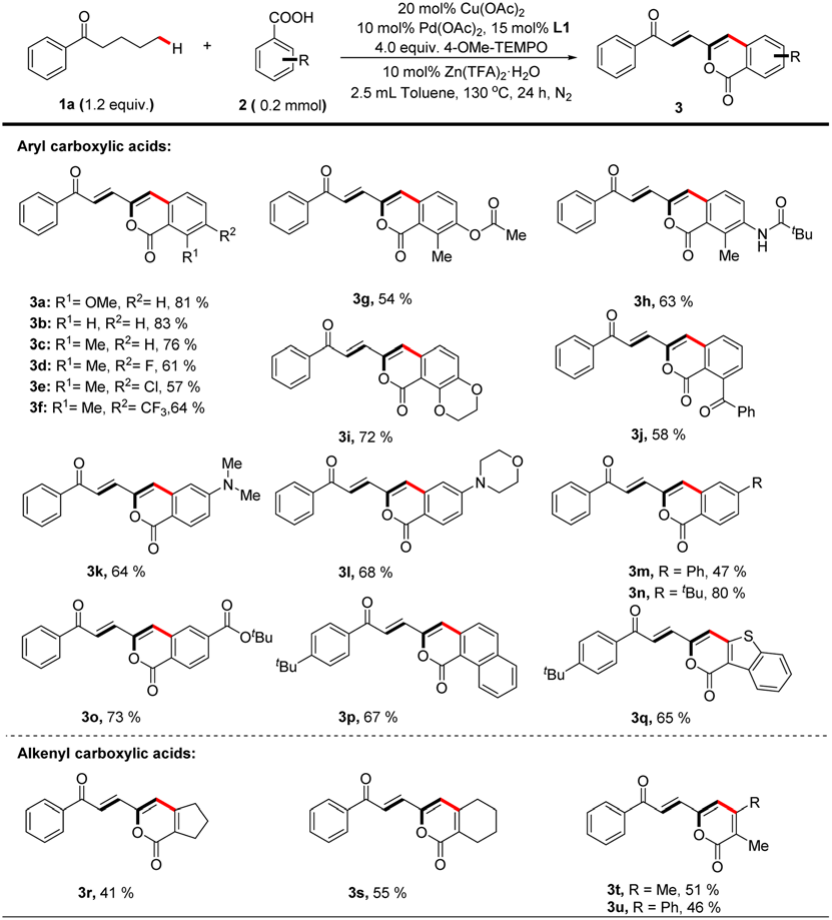

a Reaction of 1a (0.24 mmol, 1.2 equiv.) with 2a (0.2 mmol) is conducted at 130 °C in toluene (2.5 mL) for 24 hours, all isolated yields.

Next, we explored the scope of saturated ketones for the targeted reaction (Table 3). A broad range of ketones were found to perform well for this transformation. Valerophenones with different substituents at the 4-position of the phenyl ring all underwent the desired convention smoothly to give the corresponding products in good yields, and variation of the *para*-substituents of these valerophenones did not significantly affect the reaction outcomes (4a–k). Valerophenones containing multi-substituted phenyl rings were also suitable (4q–s). When alkyl vinyl ketone was employed, the targeted arylation still occurred on the alkyl moiety (4n). Other aromatic rings, such as benzothiophene (4l), benzofuran (4m), naphthalene (4o), and pyrene (4p) could also be tolerated, affording fluorescent materials with potentially interesting extended-π conjugated skeletons that are difficult to prepare^28^ As exemplified in the cases of 4t–4v, dialkyl ketones were also suitable substrates to afford alkenylated isocoumarin products in reasonable yields. Particularly noteworthy is that the dehydrogenative desaturation reaction occurred in both alkyl chains of *n*-butyl isopropyl ketone but the δ-arylated polyenone was isolated as a sole product (4u), likely because of the steric factor on the isopropyl side.

**Table 3:** The scope of ketones for bimetallic Cu–Pd catalyzed remote γ, δ-C(sp^3^)–H functionalization of aliphatic acyclic ketones^a^

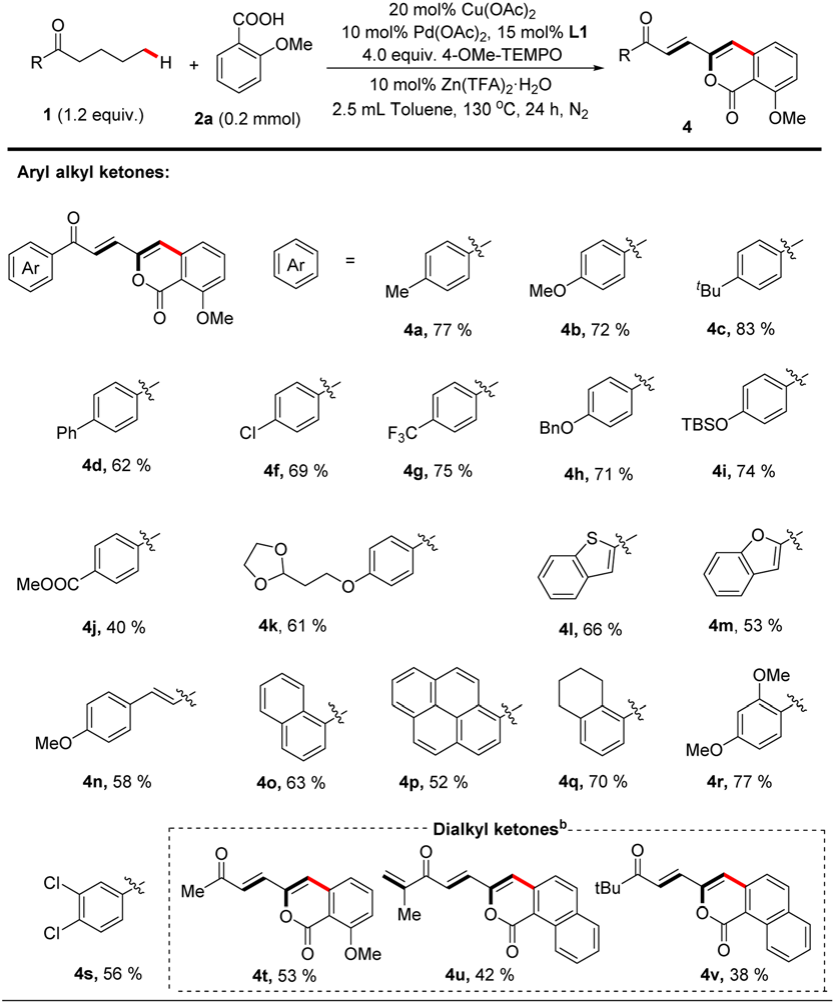

a Reaction of 1 (0.24 mmol, 1.2 equiv.) with 2a (0.2 mmol) is conducted at 130 °C in toluene (2.5 mL) for 24 hours, all isolated yields.

b 1 (0.6 mmol, 3.0 equiv.) with 2 (0.2 mmol).

**Scheme 2:**
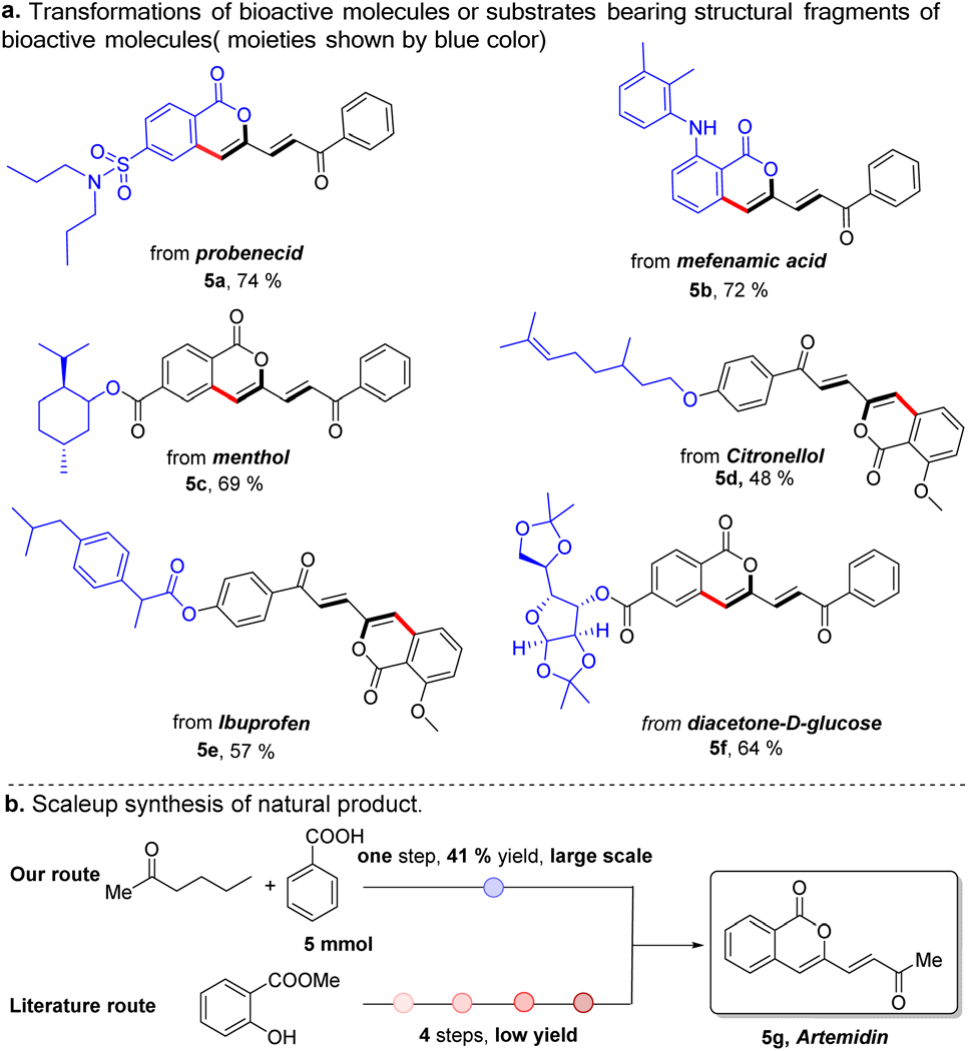
Synthetic application of our protocol.

In the last decade, late-stage modification has emerged as a promising toolbox that enables medicine chemists to rapidly diversify natural products and drugs by installing various functional groups onto target molecules to change their molecular properties. As a further demonstration of our strategy, we applied this protocol to the late-stage diversification of natural products, pharmaceuticals and derivatives (Scheme 2). In this context, the syntheses of multiple functionalized ketones starting from probenecid (5a), mefenamic acid (5b), and other complicated coupling partners derived from menthol (5c), citronellol (5d), ibuprofen (5e), and glucose (5f) were achieved, demonstrating the excellent functional group tolerance and practicability of this method. Our Pd/Cu catalyzed method also enabled one-step large scale synthesis of artemidin (5g) in 41% yield using commercially available starting materials (Scheme 2B). In sharp contrast, this natural product was previously synthesized by way of a four-step synthetic route starting from methyl salicylate.^29^ yield using commercially available starting materials (Scheme 2B).

Notably, further investigation of the substrate scope for this reaction demonstrated that our protocol also enabled 1-aryl-heptan-1-ones to undergo functionalization at remoter ε, ζ carbons *via* multiple successive dehydrogenations and afford targeted dienone-substituted isocoumarin products albeit in moderate yields (Table 4). The highly conjugated structures of dienone-substituted isocoumarin products were unambiguously established by single crystal X-ray diffraction analysis (7c) and NMR spectroscopy technologies. The prevalence of conjugated triene subunits in bioactive compounds further highlights the merit of our protocol since it opens a new avenue for direct construction of highly functionalized conjugated triene structures from simple 1-aryl-heptan-1-ones.

**Table 4:** bimetallic Cu–Pd catalyzed remote ε, ζ-C(sp^3^)–H functionalization of aliphatic acyclic ketones^a^

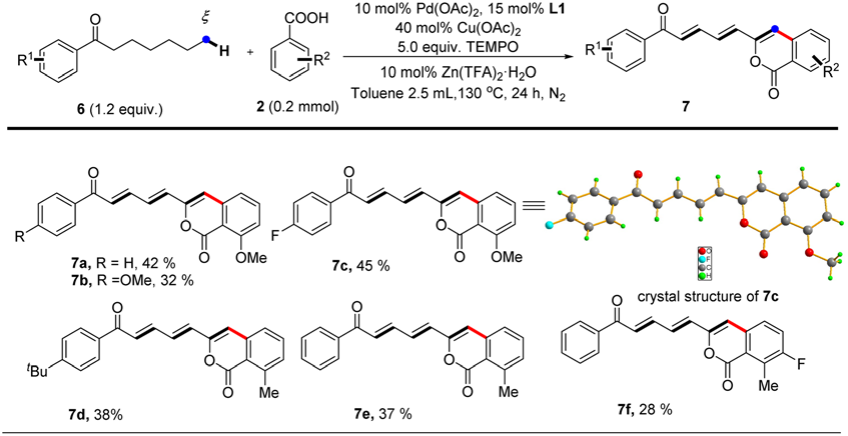

a Reaction of 1 (0.24 mmol, 1.2 equiv.) with 2a (0.2 mmol) is conducted at 130 °C in toluene (2.5 mL) for 24 hours, all isolated yields.

**Scheme 3:**
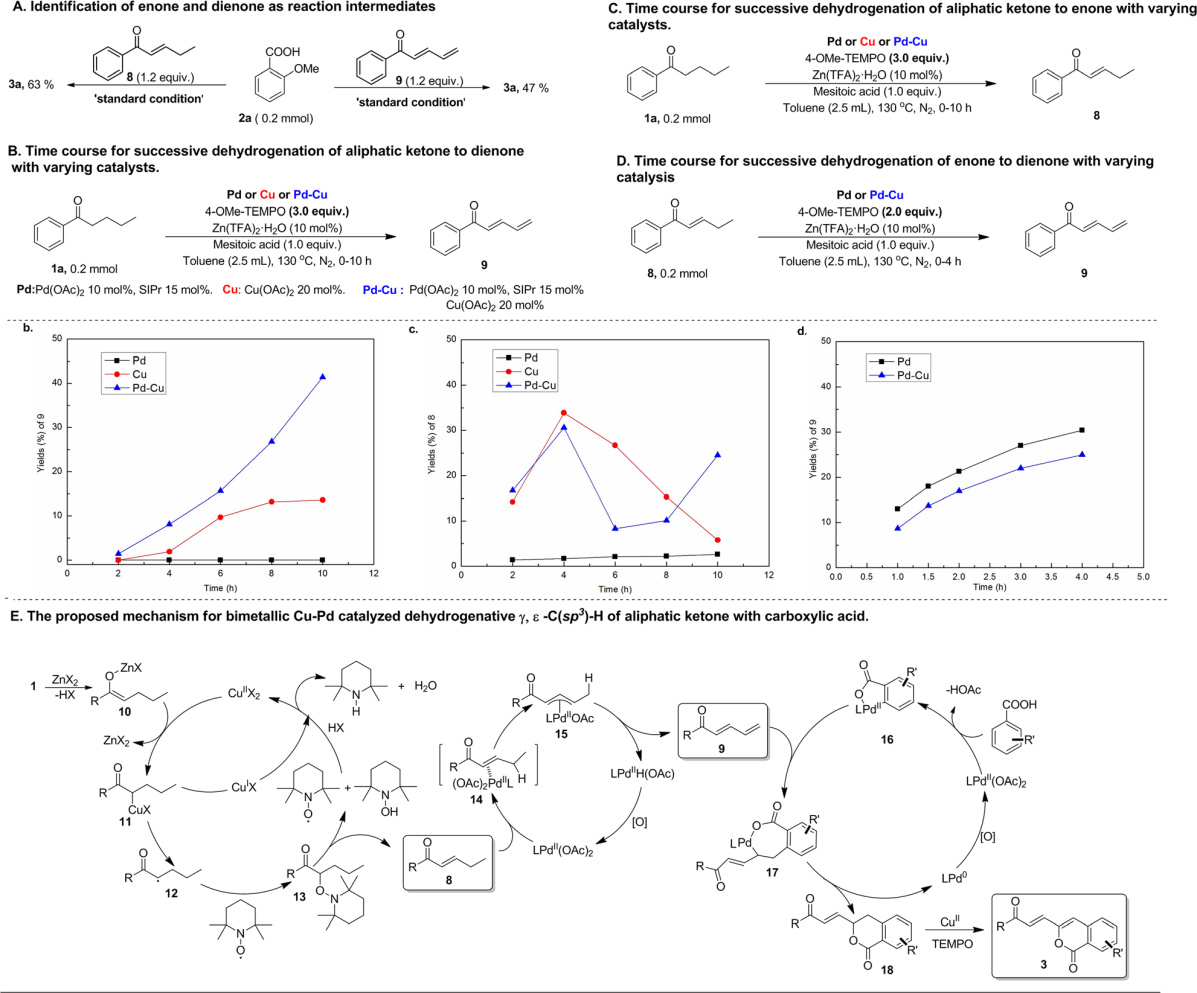
Mechanistic studies on bimetallic Cu–Pd catalyzed dehydrogenative γ, δ-C(sp^3^)–H functionalization of aliphatic acyclic ketones.

Mechanistic studies were performed to obtain insights into this bimetallic Cu–Pd catalyzed multiple C(sp^3^)–H functionalizations of aliphatic acyclic ketones. Both enone (8) and dienone (9) were used as reactants to react with aryl carboxylic acid under conditions similar to the standard reaction ones (Scheme 3A). Both reactions were observed to produce the same product as that resulting from the reaction of the corresponding aliphatic ketone, namely alkenylated-isocoumarin (3a), indicating that this bimetallic Cu–Pd-catalyzed reaction should go through enone and dienone intermediates that stem from successive dehydrogenation of aliphatic acyclic ketones. In view of the fact that the high efficiency of successive dehydrogenation-triggered multiple C(sp^3^)–H functionalization of aliphatic ketones in this bimetallic Cu–Pd catalyzed reaction contrasts sharply with the extremely low efficiency in previously reported Cu-catalyzed successive dehydrogenation of terminally unsubstituted aliphatic ketones, the effect of the bimetallic Cu–Pd catalyst system on the successive dehydrogenation of aliphatic ketones was investigated by comparing with the performance of monometallic catalysts. As shown by the time courses for the successive dehydrogenation of aliphatic ketone 1a to dienone with varying catalysts (Scheme 3B), the bimetallic Cu–Pd catalyst system provided a higher initial rate and a higher yield than the monometallic Cu catalyst, while the monometallic Pd catalyst did not provide dienone at all. Meanwhile, the time courses for α, β-dehydrogen to generate enone intermediates were also examined in these experiments conducted for successive dehydrogenation reactions. As shown in Scheme 3C, both the bimetallic Cu–Pd catalyst system and monometallic Cu catalyst gave rise to wave-like time courses for the formation of enone *via* α, β-dehydrogen of aliphatic ketone 1a, consistent with enone playing the active intermediate role in the successive dehydrogenation towards forming dienone. The difference in the time-course wave-pattern between the Cu–Pd bimetallic catalyst and the Cu catalyst implied that Pd species might influence Cu-catalyzed ketone α, β-dehydrogenation to some extent, though using the Pd catalyst alone gave only a trace of enone in this reaction process. γ, δ-Dehydrogenation of enone to dienone was accessible with the catalysts in question (Scheme 3D), suggesting that the successive dehydrogenation of aliphatic ketone to dienone should go through the enone intermediate. Investigating the time course for this transformation revealed that the monometallic Pd catalyst was almost as active as the bimetallic Cu–Pd catalyst. These experimental results offered the evidence to support the bimetallic Cu–Pd synergistically catalyzed successive dehydrogenation of aliphatic ketones into dienone, in which α, β-dehydrogenation was promoted by the Cu catalyst and γ, δ-dehydrogenation of the resulting enone to dienone was mainly promoted by the Pd catalyst.

On the basis of mechanistic studies, we proposed a plausible reaction pathway for the bimetallic Cu–Pd catalyzed multiple C(sp^3^)–H functionalizations of aliphatic ketone with aryl carboxylic acids, which merges three catalytic cycles, that is, Cu-catalyzed α, β-dehydrogenation of aliphatic ketone to enone, Pd-catalyzed γ, δ-dehydrogenation of enone to dienone, and Pd-catalyzed carboxyl-directed *ortho*-C–H alkenylation of aryl carboxylic acid with the resulting dienone (Scheme 3E). The Cu-catalyzed α, β-dehydrogenation of aliphatic ketone is initiated by transmetallation between Cu(OAc)_2_ and the Zn-enolate complex (10) *in situ* generated from ketone and Lewis-acid Zn(TFA)_2_. Upon formation of the Cu-enolate complex (11) *via* this transmetallation, hemolysis of the Cu–C bond in Cu-enolate complex 11 occurs to form the α-carbon-centered ketone radical (12) that is intercepted by TEMPO to give α-TEMPO-substituted ketone (13). Then, TEMPOH-elimination of α-TEMPO-substituted ketone, which is assisted by another TEMPO molecule, gives rise to the formation of enone (8) with the release of TEMPOH. Finally, the Cu(i) species is oxidized by TEMPO or TEMPOH to regenerate the Cu(ii) catalyst. The electron-withdrawing carbonyl group of enone increases the acidity of the γ-C(sp^3^)–H bond, thus activating the enone γ-C(sp^3^)–H bond towards Pd-mediated allylic C(sp^3^)–H cleavage *via* CMD mode to generate an allylic Pd intermediate. In light of this, Pd-mediated cleavage of the γ-C(sp^3^)–H bond occurs to form the allylic Pd intermediate (15), followed by β-H elimination of the resulting allylic Pd intermediate 15 to deliver dienone (9) and the Pd-hydride complex. Pd-hydride goes through deprotonation and oxidation to regenerate Pd(ii) species for completion of this catalytic cycle. Meanwhile, carboxyl-directed *ortho*-C(sp^2^)–H palladation of aryl carboxylic acid takes place to generate the cyclopalladation intermediate (16). The terminal double-bond of dienone 9 inserts into the Pd–C bond of 16 to form a seven-membered cyclopalladation intermediate (17) that undergoes intramolecular reductive elimination for C–O bond formation to construct a six-membered lactone intermediate (18) with the formation of Pd(0) species. Finally, the resultant lactone intermediate (18) undergoes Cu-catalyzed dehydrogenation-desaturation at the positions γ and δ to the carbonyl group with TEMPO as an oxidant to afford an alkenylated-isocoumarin product (3), since the lactone intermediate (18) containing the terminally arylated enone motif is active towards Cu/TEMPO mediated remote dehydrogenation desaturation. In the catalytic cycle for Pd-catalyzed coupling of aryl carboxylic acid with dienone, it is unlikely that the seven-membered cyclopalladation intermediate 17 undergoes β-H elimination to form *ortho* dienone-substituted aryl carboxylic acid and subsequent metal-promoted intramolecular nucleophilic addition of carboxyl to the terminal double bond since this process should occur preferentially at the electrophilic ε-carbon of dienone to give a five-membered lactone. The soft, strongly electron-donating *N*-heterocycle carbene ligand combines preferentially with softer Pd species and therefore promotes oxidation of Pd(0) species by weak oxidant TEMPO to Pd(ii) species in the Pd(ii)/Pd(0) catalytic cycle.^24,30^

## Conclusions

In summary, an efficient bimetallic Cu–Pd catalyzed method has been developed to achieve dehydrogenation desaturation-triggered coupling reactions of aliphatic acyclic ketones with aryl carboxylic acids, offering a concise approach to direct syntheses of functionally condensed alkenylated isocoumarin products in generally good yields from readily available reactants. This bimetallic Cu–Pd catalyzed, dehydrogenation-triggered coupling reaction features multiple functionalizations of the C(sp^3^)–H bonds at remote positions γ, δ or ε, ζ to carbonyl groups of aliphatic ketones, complementing the existing methods for C(sp^3^)–H functionalization in terms of the regioselectivity of the reaction and the factors controlling regioselectivity. This method enjoys a high functional group tolerance as exemplified by conversion of complicated bioactive molecules and can be extended to alkenyl carboxylic acids. As revealed by mechanistic studies, the success achieved in developing this efficient bimetallic-catalyzed method is ascribed to the synergistic bimetallic Cu–Pd catalysis for successive dehydrogenation desaturation of aliphatic acyclic ketones, which exhibits high efficiency and overcomes the long-standing challenge posed by the successive dehydrogenation desaturation of terminally unsubstituted aliphatic ketones to dienones or polyenones. Given the versatile reactivity of di- and poly-enones and the high functional group tolerance of this bimetallic-catalyzed successive dehydrogenation, huge effort is underway in our group to develop diverse methods for dehydrogenation-triggered remote C(sp^3^)–H functionalizations of aliphatic ketones *via* bimetallic synergistically catalyzed successive dehydrogenation.

## Data availability

The authors declare that the data supporting the findings of this study are available within the paper and the ESI,† as well as from the authors upon request.

## Author contributions

H. L. and W. S. conceived the project; H. L. performed the experiments and analyzed the data; C. Y. and S. L. prepared some starting materials; H. T. discussed the results; P. L. characterized the X-ray structures of 7c; H. L., J. C., and W. S. prepared this manuscript.

## Conflicts of interest

There are no conflicts to declare.

## Supplementary Material

SC-013-D2SC05370E-s001
